# Initiatives of 11 Rural Appalachian Cancer Coalitions in Pennsylvania and New York

**Published:** 2006-09-15

**Authors:** Eugene J Lengerich, Brenda C Kluhsman, Marcyann Bencivenga, Erik Lehman, Ann J Ward

**Affiliations:** The Pennsylvania State University, Department of Health Evaluation Sciences. Dr Lengerich is also affiliated with the Penn State Cancer Institute, Hershey, Pa; Department of Health Evaluation Sciences, College of Medicine, The Pennsylvania State University, Hershey, Pa; Department of Health Evaluation Sciences, College of Medicine, The Pennsylvania State University, Hershey, Pa; Department of Health Evaluation Sciences, College of Medicine, The Pennsylvania State University, Hershey, Pa; Community Cancer Control, Research, and Education, Lemont, Pa

## Abstract

**Introduction:**

Local coalitions combine the knowledge, expertise, and resources of many individuals and organizations to improve community health. This article describes data from 11 rural cancer coalitions in Pennsylvania and New York collected through a model-based data system.

**Methods:**

The coalition data collection system was adapted from a conceptual model designed to monitor the activities and impact of cardiovascular disease coalitions. Community Coalition Action Theory was used during implementation and validation of the data system. Primary components of the data system were organizational capacity, process, and outcome/impact.

**Results:**

From 2002 to 2004, the 11 coalitions conducted 1369 initiatives, including 1147 (83.8%) interventions and 222 (16.2%) development activities. Among interventions, 776 (56.7%) were outreach only, 158 (11.5%) education only, 117 (8.5%) outreach and education, and 96 (7.0%) screening. Differences in the distribution of initiatives by coalition, cancer site, and target audience were statistically significant (*P* < .05). The majority of interventions focused on colorectal (37.0%) and breast (32.9%) cancer. Target groups included women (71.3%), rural residents (32.6%), survivors (21.8%), and low-income (21.8%) individuals. Although not statistically significant, an observed 3-year trend was shown for decreased outreach interventiions and increased education and screening interventions. In total, 1951 of 3981 individuals who were offered a cancer screening (49%) completed screening, and 15 sustainable community changes were documented.

**Conclusion:**

This study reports the initiatives and impact of 11 rural community cancer coalitions over a 3-year period. This study estimates the mix of development activities and community interventions, against which this coalition network and others may be compared.

## Introduction

Local coalitions combine the knowledge, expertise, and resources of multiple individuals and organizations to improve community health (1,2). However, measurement of the process and impact of coalitions is difficult because of the complexity of coalitions and because traditional methods are poorly suited for capturing information on coalitions (3). Program planning, implementation, and monitoring processes that are based in theory are more likely to succeed than those processes developed without a theoretical perspective (4). Therefore, systems to monitor and evaluate coalition processes and impact should be based on sound theoretical and conceptual frameworks. To date, few theory-based coalition data collection and evaluation systems have been reported in the literature.

Methods to better understand and measure the processes and impact of community coalitions include performance monitoring (5,6); ecological assessment (7); mixed-methods evaluation (8,9); and process evaluation, monitoring, and feedback (10-14). Early conceptual models developed to guide the capacity-building of coalitions include the PRECEDE–PROCEED Planning Model (15,16) and the Planned Approach To Community Health (PATCH) (17). In addition, researchers at the University of Kansas developed a data system and toolbox (18) based on a logic model approach to community change to enhance the potential of community health coalitions to address various disease conditions (10).

More recently, Butterfoss and Francisco (19) recommended measurement of health status and community change in coalition-based studies in addition to measurement of coalition capacity and sustainability. The Community Coalition Action Theory (CCAT) model identifies internal coalition factors and processes that lead to implementation of strategies and community change (20) and thereby provides a method to assess coalition efforts. Additionally, the Reach, Efficacy, Adoption, Implementation, and Maintenance (RE-AIM) evaluation framework uses quantifiable evaluation measures through each phase of research (21,22) to increase the likelihood of program adoption by coalitions in applied settings (23).

The potential for community coalitions as cancer prevention and control agents has been reported previously (24-29). In a recent controlled community intervention study, Ward et al (30) found that nine cancer coalitions in 13 rural Appalachian counties of Pennsylvania and New York recruited almost three times as many local organizations to a cancer education intervention than did matched control counties that did not have a cancer coalition.

As cancer coalitions increasingly engage in protocol-driven research, activities, and interventions, they will need theory-based data systems to monitor processes and assess impact. Coalitions will use these systems and the data that result from them to help describe intervention processes and impact, develop community cancer plans, and prepare funding proposals. Although recent advances have been made in the development of Web-based applications for public health education and data collection (31-33), data collection systems designed specifically for community-based cancer coalitions have not been reported previously. The purpose of this paper is to describe a model-based data system and the data of 11 rural cancer coalitions in Pennsylvania and New York collected from 2002 through 2004. The coalitions were part of the Appalachia Cancer Network (ACN) (2000–2005), an academic–community partnership for community-based participatory research in cancer prevention and control (34,35). The coalitions were formed during the Appalachia Leadership Initiative on Cancer (1992–2000) and currently are part of the Appalachia Community Cancer Network (2005–2010). This study included the nine coalitions that participated in the Ward et al (30) study.

## Methods

The coalition data collection system was initially adapted from a conceptual model designed to monitor the activities and impact of cardiovascular disease coalitions (10). The CCAT model (20) provided the conceptual framework for coalition development activities and interventions. Data collected from 2002 through 2004 from 11 coalitions were included in the analysis. One coalition had recorded data for only the first 1.5 years of the study period due to loss of field staff. The data collection system contained three primary components (Figure).

**Figure 1:**
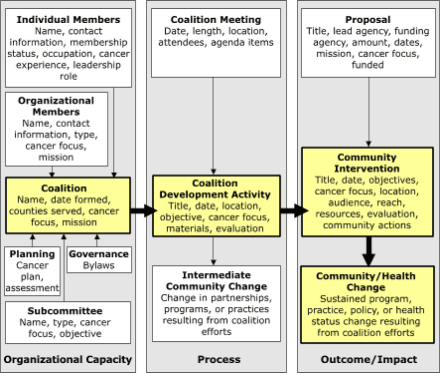
Coalition conceptual model, 11 rural Appalachian coalitions in Pennsylvania and New York, 2002–2004. Adapted from: Francisco et al (10).

**Table:** 

This diagram is a coalition conceptual model that shows 11 rural Appalachian coalitions in Pennsylvania and New York. It is divided into three large rectangular sections; each is labeled at the bottom. The first one is labeled “Organizational Capacity,” which points right to the section labeled “Process,” which points further right to the section labeled “Outcome/Impact.”
The Organizational Capacity section contains two boxes at the top that read 1) “Individual Members: Name, contact information, membership status, occupation, cancer experience, leadership role,” and 2) “Organizational Members: Name, contact information, type, cancer focus, mission.” There are three boxes at the bottom that read 1) “Planning: Cancer plan, assessment, 2) “Governance: Bylaws,” and 3) “Subcommittee: Name, type, cancer focus, objective.” The boxes at the top and bottom point to a box in the center that reads, “Coalition: Name, date formed, counties served, cancer focus, mission.”
The Process section contains a box at the top that reads, “Coalition Meeting: Date, length, location, attendees, agenda items.” This box points down to a box that reads, “Coalition Development Activity: Title, date, location, objective, cancer focus, materials, evaluation” which points down to a box at the bottom that reads, “Intermediate Community Change: Change in partnerships, programs, or practices resulting from coalition efforts.”
The Outcome/Impact section begins with a box at the top that reads, “Proposal: Title, lead agency, funding agency, amount, dates, mission, cancer focus, funded.” This box points down to another box that reads, “Community Intervention: Title, date, objectives, cancer focus, location, audience, reach, resources, evaluation, community actions.” This one points down to a final box that reads “Community/Health Change: Sustained program, practice, policy, or health status change resulting from coalition efforts.”

*Organizational capacity* included data on each coalition, including individual and organizational members, geographic service area, mission and focus, cancer control plans, governance, and subcommittees.

*Process* included coalition development activities, defined as "a planned gathering of some or all coalition members primarily intended to educate, equip, or enlarge coalition membership." The primary audience for development activities was current or potential coalition members. Examples of development activities included member recruitment, training and recognition, and community needs assessment. Data items included meeting information, development activity, and intermediate community change. Unlike Francisco et al (10), our model included intermediate community change, such as development of partnerships with new organizations or a change in practices or policies of partner organizations.

*Outcome/Impact* included intermediate outcomes such as community interventions, funded proposals, and impact such as community changes and cancer screenings. Community interventions were defined as “planned events sponsored or cosponsored by the coalition and primarily intended to directly change behavior, detect risk or disease, or educate persons who are not in the coalition (nonmembers).” Community interventions were the primary mechanism by which the coalition fulfilled its mission of cancer control. Similar to Francisco et al (10), community change met the following criteria: 1) included members outside of the coalition; 2) was related to the coalition’s mission; 3) was facilitated by coalition members; 4) generated new or modified programs, policies, or practices that were likely to occur without coalition influence in the future; and 5) occurred (was not just planned).

The cancer coalition data system was a secure, password-protected, Web-based data application designed and beta tested by programming and field staff. Field staff received approximately 40 hours of initial training in data collection, coding, and data entry, with continued training for 3 months to maximize data validity and reliability. A training manual, data coding criteria, and system operating procedures were also developed.

Data were collected by northern ACN field staff on nine distinct data collection forms: coalition information, coalition meeting, coalition meeting attendees, coalition individual member, coalition organization member, subcommittee, coalition development activity, community intervention, and proposal/fundraiser. Identical computer screen forms were developed for electronic data entry. Field staff had primary responsibility for completing initial data entry based on their attendance at coalition meetings and collection of follow-up data related to coalition initiatives. Quality assurance was provided by the project director and data managers.

For analysis purposes, coalition initiatives were defined as either a coalition development activity or a community intervention. The objectives of the community interventions were determined and recorded; each intervention could have more than one objective. Objectives were used to group interventions into education-only, outreach-only, education and outreach, and screening interventions.

Statistical analyses of coalition initiatives were completed with SAS version 9.1 (SAS Institute, Cary, NC). Because there were multiple observations from each coalition within each type of initiative, a generalized estimating equations model was used to test for a temporal trend in the proportion of each initiative. A Pearson Χ^2^ test was used within each type of initiative to test for differences in the proportions of the initiatives between coalitions because there was only one observation per coalition per initiative. Generalized estimating equations were used to test for differences between proportions of the rows of cancer focus or audience within each type of initiative because there were potentially multiple observations from each coalition within each type of initiative. The significance level was set at 0.05.

This research was approved by the institutional review board of The Pennsylvania State University. Coalition members demonstrated consent by completion of individual member forms.

## Results

From 2002 to 2004, the 11 Pennsylvania and New York coalitions conducted 1369 initiatives, including 222 (16.2%) development activities and 1147 (83.8%) interventions (Table 1). Of the interventions, 776 (56.7%) were outreach only, 158 (11.5%) education only, 117 (8.5%) outreach and education, and 96 (7.0%) screening. Outreach-only interventions decreased from two in every three interventions to one in every two interventions, whereas the number and percentage of screening interventions (and education interventions generally) increased each year; neither trend was statistically significant.

The median number of initiatives per coalition was 112 (range, 18–274) (Table 2). Except in two cases, each coalition completed at least one of each type of initiative during the 3-year study period. The median number of screening interventions was four (range, 0–50). The distribution of the specific coalitions by each type of initiative was statistically significant (*P* < .001).

The primary cancer focus of slightly more than one third (37.0%) of the interventions was colorectal cancer (n = 423) and another one third (32.9%) was breast cancer (n = 376) (Table 3). Among screening interventions, 51 (50.5%) were focused on breast cancer, and 28 (27.7%) were focused on colorectal cancer. The distribution of primary cancer focus by each type of intervention was statistically significant (*P* < .001 for outreach-only, education-only, and outreach and education interventions; *P* = .006 for screening interventions).

Of the 1164 interventions that targeted an age group, almost half (47.2%) were targeted to individuals aged 50 to 64 years with another one third (34.9%) targeted to individuals aged 65 years or older (Table 4). Approximately 71% of the 567 interventions that targeted a specific sex were targeted to women (n = 404). Of the 472 interventions that targeted a particular audience, 154 (32.6%) were targeted to rural residents. Almost a quarter of the interventions each were targeted to survivors (21.8%) or low-income individuals (21.8%). Of the 63 interventions that targeted health care providers, half (52.4%) were targeted to nurses. Among the 91 screening interventions that targeted a specific age group, 79 were targeted to individuals aged 50 years and older. Among the 80 screening interventions that targeted a specific sex, 56 were targeted to women.

From 2002 to 2004, of 3981 community residents reached through screening interventions, 1951 (49%) completed cancer screening (data not shown). In addition, 15 sustainable community changes were reported by three of the 11 coalitions. The foci of these community changes were colorectal cancer (n = 11), breast and cervical cancer (n = 4), tobacco (n = 1) and prostate cancer (n= 1). These sustainable community changes included primary care clinics that signed contracts to offer free and low-cost breast and cervical cancer screening services to underinsured individuals; clinic space made available for screening services for underinsured individuals; funding provided for cancer screening among underinsured individuals; and restaurants that became smoke-free.

## Discussion

From 2002 to 2004, the 11 rural cancer coalitions in Pennsylvania and New York completed 1369 coalition initiatives. Of these initiatives, six of every seven were community interventions with one in seven being coalition development activities. Development activities are necessary for long-term sustainability of a cancer coalition because it is through these activities that coalitions recruit, educate, and equip coalition members. However, the appropriate ratio of community interventions to development activities for effective, efficient, and sustainable cancer coalitions has not been estimated. The ratio of community interventions to development activities for the 11 coalitions in this study was substantial and ranged from 1.3:1.0 to 16.8:1.0; the overall ratio of community interventions to development activities for this study was 5.2:1.0.

Within community interventions, the number and percentage of screening interventions (and education interventions generally) increased during the study period, whereas the number and percentage of outreach-only interventions decreased during the study period. In fact, outreach-only activities decreased from two in every three interventions to one in every two interventions. This appropriate increase in proportion of interventions may be partially attributed to the participatory partnership between the coalitions and the academic researchers. The research team provided monthly technical assistance, support, and training that strengthened coalition efforts, focus, and direction. We are encouraged by these changes because education and screening interventions are more likely to have a direct impact on cancer prevention and control than are outreach-only interventions. Additional years of data may establish that the temporal trend for these 11 coalitions was statistically significant.

It is difficult to associate specific outcomes, such as a community change in cancer risk, to outreach-only interventions because outreach-only interventions tend to be nonspecific and difficult to measure. Interventions that are more individualized and easier to measure would include those with an education or screening objective. However, outreach-only interventions may be an important component in a comprehensive mix of coalition interventions. Although the ideal ratio of outreach-only to targeted interventions is not known, the range for the 11 individual coalitions was 0.5:1.0 to 9.0:1.0, and the ratio for all coalitions was 2.1:1.0.

The measures and observed results of coalition effectiveness in this study, including the increasing trend in interventions, the substantial number of completed screenings, and documented community changes, are indicators of the success of the 11 coalitions over the 3-year study period. The CCAT model (20) posits that the combined resources and intervention strategies of coalition members and their partners can improve health outcomes and lead to sustainable community change. The 11 coalitions and their academic partners in this study were able to achieve more through their participatory partnership than any one of the coalitions or the research team alone could have achieved, which is a principal tenet of community-based participatory research (36). Interventions were possible because of the long history and trusted relationships between the coalitions and their communities. The researchers added scientific rigor and a system for documenting processes and impact of the coalitions' development activities and interventions. Thus, the partnership of coalitions and academic researchers greatly enhanced the potential to reduce the cancer burden in this rural Appalachian population.

This study is limited in several aspects. First, the validity and reliability of this coalition data system have not been formally tested. However, extensive training of field staff, their ongoing use of data manuals, as well as quality-control procedures and oversight of the project director improved reliability and reduced potential misclassification bias. In addition, this data system was developed from an established community-based intervention model. Second, a primary impact, community change, was infrequently reported. Many coalitions rarely focused on community change as a primary objective and focused instead on community education, screening recruitment, and cancer prevention. Anecdotal reports indicate that community changes occurred more frequently than were recorded in the data system. This is partially because a substantial amount of time frequently lapsed between an intervention and the resulting sustainable community change. This time delay may be responsible for the lack of recorded data on community change in the data system. Therefore, we believe that we have underestimated the true number of community changes that resulted, at least in part, from these 11 rural coalitions. Third and finally, because the population was mostly rural and white, the results cannot be generalized to other populations. Further studies are needed to establish external validity to nonrural and diverse racial and ethnic groups.

Despite these limitations, this study has several strengths. First, this study documents the activities and impact of 11 community cancer coalitions over a 3-year study period. These coalitions have been in existence for more than a decade, so these results represent coalitions with an extensive history. More importantly, these data were captured through a model- and Web-based approach for coalition-driven, community interventions in cancer prevention and control. The Web-based data system is mutually beneficial to both researchers and communities, particularly those in rural, less accessible areas. By allowing remote data entry by regional field staff who reside near coalitions that are dispersed across a wide geographical area, the data system enhances timely submission of coalition data, provides  technical assistance and communication with the coalitions, and reduces travel costs. In turn, coalitions receive data for strategic planning, new member recruitment, and program evaluation and report these data back to their communities. The Internet has been used to assist women in rural areas with day-to-day management of breast cancer (37), provide electronic support groups for breast cancer patients  (38), and improve quality of life for low-income women with breast cancer (39). However, to our knowledge, this data collection system is relatively unique for evaluating community-based cancer prevention and control initiatives. Finally, this study provided estimates of a mix of various development activities and community interventions, aggregated and compared across 3 years, against which comparisons with this network or other networks of community coalitions may be made. Analyses of future coalition outcomes examined in comparison with the current study will help establish the long-term sustainability of these coalitions and provide recommendations for an appropriate mix of coalition development activities and interventions that achieve measurable reduction in cancer burden for underserved populations.

This study demonstrates that community cancer coalitions in Pennsylvania and New York reached rural residents through cancer prevention and early detection education and screening interventions. Additional research is needed to determine the appropriate mix of development activities and community interventions for coalitions to achieve their cancer prevention and control goals. In addition, evidence-based interventions are needed for coalitions to meet the cancer-related needs of specific populations. Finally, improved measurement of cancer screenings and community change will help coalitions document progress toward their overall goal of reducing cancer disparities in rural Appalachia.

## Figures and Tables

**Table 1:** Coalition Initiatives by Type and Study Year, 11 Rural Appalachian Coalitions in Pennsylvania and New York, 2002–2004^a^

**Study Year**	**Type of Initiative^b^**					**Total, No. (%)**

	Development**, No. (%)**	**Outreach Only, No. (%)**	**Education Only, No. (%)**	**Outreach and Education, No. (%)**	**Screening, No. (%)**
2002	56 (12.2)	301 (65.7)	46 (10.0)	33 (7.2)	22 (4.8)	458 (33.5)
2003	90 (20.0)	248 (55.0)	40 (8.9)	38 (8.4)	35 (7.8)	451 (32.9)
2004	76 (16.5)	227 (49.3)	72 (15.7)	46 (10.0)	39 (8.5)	460 (33.6)
**Total**	222 (16.2)	776 (56.7)	158 (11.5)	117 (8.5)	96 (7.0)	1369 (100.0)

NA indicates not applicable.

a One coalition recorded data for only half of study period.

b Test for trend using generalized estimating equations: *P* = .17 for development initiatives, *P* = .12 for outreach only, *P* = .36 for education only, *P* = .46 for outreach and education, and *P* = .09 for screening.

**Table 2:** Coalition Initiatives by Coalition and Type, 11 Rural Appalachian Coalitions in Pennsylvania and New York, 2002–2004

**Coalition** a	**Type of Initiative^b^**					**Total, No. (%)**

	**Development, No. (%)**	**Outreach Only, No. (%)**	**Education Only, No. (%)**	**Outreach and Education, No. (%)**	**Screening, No. (%)**	
1	10 (5.6)	140 (78.7)	25 (14.0)	1 (0.6)	2 (1.1)	178 (13.0)
2	38 (13.9)	124 (45.3)	53 (19.3)	9 (3.3)	50 (18.2)	274 (20.0)
3	40 (28.2)	71 (50.0)	2 (1.4)	22 (15.5)	7 (4.9)	142 (10.4)
4	28 (43.8)	14 (21.9)	8 (12.5)	0 (0.0)	14 (21.9)	64 (4.7)
5	14 (8.4)	111 (66.5)	6 (3.6)	32 (19.2)	4 (2.4)	167 (12.2)
6	30 (27.8)	58 (53.7)	15 (13.9)	3 (2.8)	2 (1.9)	108 (7.9)
7	2 (11.1)	5 (27.8)	2 (11.1)	7 (38.9)	2 (11.1)	18 (1.3)
8	22 (18.6)	42 (35.6)	27 (22.9)	20 (16.9)	7 (5.9)	118 (8.6)
9	13 (14.3)	52 (57.1)	9 (9.9)	11 (12.1)	6 (6.6)	91 (6.6)
10	13 (13.4)	69 (71.1)	8 (8.2)	5 (5.2)	2 (2.1)	97 (7.1)
11	12 (10.7)	90 (80.4)	3 (2.7)	7 (6.3)	0 (0.0)	112 (8.2)
**Total**	222 (16.2)	776 (56.7)	158 (11.5)	117 (8.5)	96 (7.0)	1369 (100.0)

a Coalition no. 6 recorded data for only half of study period.

b *P* < .001 for each type of initiative (χ^2^ test)

**Table 3:** Coalition Interventions by Primary Cancer Focus and Type, 11 Rural Appalachian Coalitions in Pennsylvania and New York, 2002–2004^a^

**Primary Cancer Focus^b^**	Type of Intervention^c^				**Total, No. (%)**

	**Outreach Only, No. (%)**	**Education Only, No. (%)**	**Outreach and Education, No. (%)**	**Screening, No. (%)**	
General	58 (43.9)	9 (6.8)	63 (47.7)	2 (1.5)	132 (11.5)
Breast	251 (66.8)	53 (14.1)	21 (5.6)	51 (13.6)	376 (32.9)
Cervical	2 (33.3)	1 (16.7)	1 (16.7)	2 (33.3)	6 (0.5)
Colorectal	331 (78.3)	51 (12.1)	13 (3.1)	28 (6.6)	423 (37.0)
Prostate	30 (48.4)	21 (33.9)	2 (3.2)	9 (14.5)	62 (5.4)
Skin	45 (54.2)	14 (16.9)	16 (19.3)	8 (9.6)	83 (7.3)
Nutrition	4 (57.1)	2 (28.6)	1 (14.3)	0 (0.0)	7 (0.6)
Tobacco	44 (80.0)	6 (10.9)	4 (7.3)	1 (1.8)	55 (4.8)
**Total**	765 (66.9)	157 (13.7)	121 (10.6)	101 (8.8)	1144^d^ (100.0)

a One coalition had recorded data for only half of study period.

b More than one primary cancer focus per intervention was possible.

c *P* < .001 for outreach-only, education-only, and outreach and education interventions; *P* = .006 for screening initiatives (test for differences in proportions using generalized estimating equations).

d No cancer focus reported for three interventions.

**Table 4:** Coalition Interventions by Designated Target Audience and Type, 11 Rural Appalachian Coalitions in Pennsylvania and New York, 2002–2004^a^

**Target Audience^b^**	Type of Intervention								**Total, No. (%)**

	Outreach Only		**Education Only**		**Outreach and Education**		**Screening**		

	**No. (%)**	***P***	**No. (%)**	***P***	**No. (%)**	***P***	**No. (%)**	***P***	
Age, y	888 (76.3)	<.001	142 (12.2)	<.001	43 (3.7)	<.001	91 (7.8)	<.001	1164 (100.0)
<18	15 (26.8)		18 (32.1)		23 (41.1)		0 (0.0)		56 (4.8)
18-39	7 (53.9)		2 (15.4)		2 (15.4)		2 (15.4)		13 (1.1)
40-49	114 (81.4)		11 (7.9)		5 (3.6)		10 (7.1)		140 (12)
50-64	430 (78.3)		51 (9.3)		7 (1.3)		61 (11.1)		549 (47.2)
≥65	322 (79.3)		60 (14.8)		6 (1.5)		18 (4.4)		406 (34.9)
Sex	382 (67.4)	.96	86 (15.2)	.70	19 (3.4)	.08	80 (14.1)	.74	567 (100.0)
Female	272 (67.3)		60 (14.9)		16 (4.0)		56 (13.9)		404 (71.3)
Male	110 (67.5)		26 (16.0)		3 (1.8)		24 (14.7)		163 (28.7)
Population group	241 (51.1)	<.001	66 (14.0)	.004	94 (19.9)	<.001	71 (15.0)	<.001	472 (100.0)
African American	2 (33.3)		1 (16.7)		1 (16.7)		2 (33.3)		6 (1.3)
Hispanic	2 (40.0)		1 (20.0)		0 (0.0)		2 (40.0)		5 (1.1)
Amish	2 (9.1)		2 (9.1)		0 (0.0)		18 (81.8)		22 (4.7)
Rural	63 (40.9)		36 (23.4)		28 (18.2)		27 (17.5)		154 (32.6)
Survivors	53 (51.5)		14 (13.6)		35 (34.0)		1 (1.0)		103 (21.8)
Low income	79 (76.7)		3 (2.9)		1 (1.0)		20 (19.4)		103 (21.8)
Caregivers	39 (50.7)		9 (11.7)		29 (37.7)		0 (0.0)		77 (16.3)
Migrant	1 (50.0)		0 (0.0)		0 (0.0)		1 (50.0)		2 (0.4)
Health care provider	11 (17.5)	.35	29 (46.0)	.01	23 (36.5)	.12	0 (0.0)	NA	63 (100.0)
Midlevel	3 (21.4)		6 (42.9)		5 (35.7)		0 (0.0)		14 (22.2)
Nurse	4 (12.1)		19 (57.6)		10 (30.3)		0 (0.0)		33 (52.4)
Physician	4 (25.0)		4 (25.0)		8 (50.0)		0 (0.0)		16 (25.4)

NA indicates not applicable.

a One coalition recorded data for only half of study period.

b More than one target audience per intervention was possible.
